# Sandwich Reconstruction Technique for Severe Glenoid Bone Loss in Reverse Shoulder Arthroplasty Using Iliac Crest Autograft and Coracoid Transfer

**DOI:** 10.1016/j.eats.2025.103846

**Published:** 2025-08-29

**Authors:** Vasileios Akrivos, Gabriel Levy, Jeanni Zbinden, Philippe Collin, Alexandre Lädermann

**Affiliations:** a Department of Orthopaedic Surgery and Musculoskeletal Trauma, Faculty of Medicine, School of Health Sciences, University of Thessalia, Larissa, Greece; b Division of Orthopaedics and Trauma Surgery, La Tour Hospital, Meyrin, Switzerland; c Faculty of Medicine, University of Geneva, Geneva, Switzerland; d CHP Saint-Grégoire, Saint-Grégoire, France; e Clinique Victor Hugo, Paris, France; f American Hospital of Paris, Neuilly-sur-Seine, France; g Division of Orthopaedics and Trauma Surgery, Department of Surgery, Geneva University Hospitals, Geneva, Switzerland

## Abstract

This Technical Note describes a sandwich grafting technique that combines a posterior tricortical iliac crest autograft with an anterior vascularized coracoid graft, secured with a full wedge-augmented baseplate. The method enhances fixation by leveraging the mechanical stability of the iliac crest graft, the biological integration of the vascularized coracoid, and the stability provided by the wedge-augmented baseplate. The screws are positioned in 2 axes to optimize stability and promote graft incorporation. This dual-graft approach addresses the limitations of single-graft techniques and augmented baseplates by restoring native glenoid anatomy and improving implant stability. The technique is particularly suited for cases with severe anterior-posterior or global glenoid bone loss, as classified by the Walch B3, C, and D categories.

The number of cases with glenoid bone loss is expected to increase in the future as a result of the increasing prevalence of prosthetic implants, longer life expectancy, and the greater functional demands of patients.^1^^,^^2^ Aseptic glenoid baseplate loosening occurs in 2.69% of cases involving osteoarthritis with bone loss, with an increased incidence of 6.8% in revision procedures involving baseplate failure. The standard baseplates may be insufficient for achieving optimal fixation in the presence of compromised bone stock, potentially leading to instability, early loosening, and implant failure.^3^, ^4^, ^5^, ^6^

Structural bone grafting during reverse shoulder arthroplasty (RSA) typically is considered when native bone coverage of the baseplate is less than 50%.^7^ Although various grafting techniques—including humeral head autografts in primary cases, tricortical iliac crest grafts, and allografts—have been proposed for glenoid bone loss, each presents limitations such as graft availability, morbidity, or insufficient mechanical integrity.^8^ In revision cases and primary cases with massive uncontained bone loss, humeral head autograft is either unavailable or insufficient, necessitating alternatives such as an iliac crest autograft or various allografts. However, iliac crest bone graft harvest is associated with a 15% donor-site morbidity rate, whereas allografts demonstrate low incorporation rates and high complication rates.^9^, ^10^, ^11^

An alternative approach for surgeons is the use of augmented components and patient-specific implants. The use of custom 3-dimensional−printed implants for managing severe glenoid bone loss in both primary and revision RSA has demonstrated significant clinical improvements in this complex patient population. However, these options are associated with greater costs.^12^ This Technical Note describes a composite "sandwich" grafting technique that combines an iliac crest autograft posteriorly with a pediculated coracoid graft anteriorly, along with a full wedge-augmented baseplate.

## Surgical Technique

### Preoperative Planning

**Fig 1:**
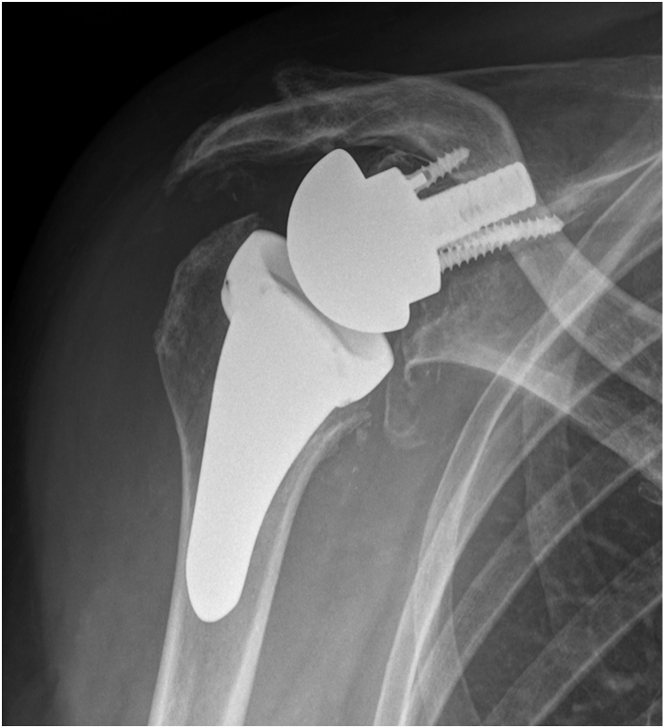
An anteroposterior radiograph of the right shoulder demonstrating loosening of the glenoid baseplate, glenoid dislocation, and possible glenoid fracture.

**Fig 2:**
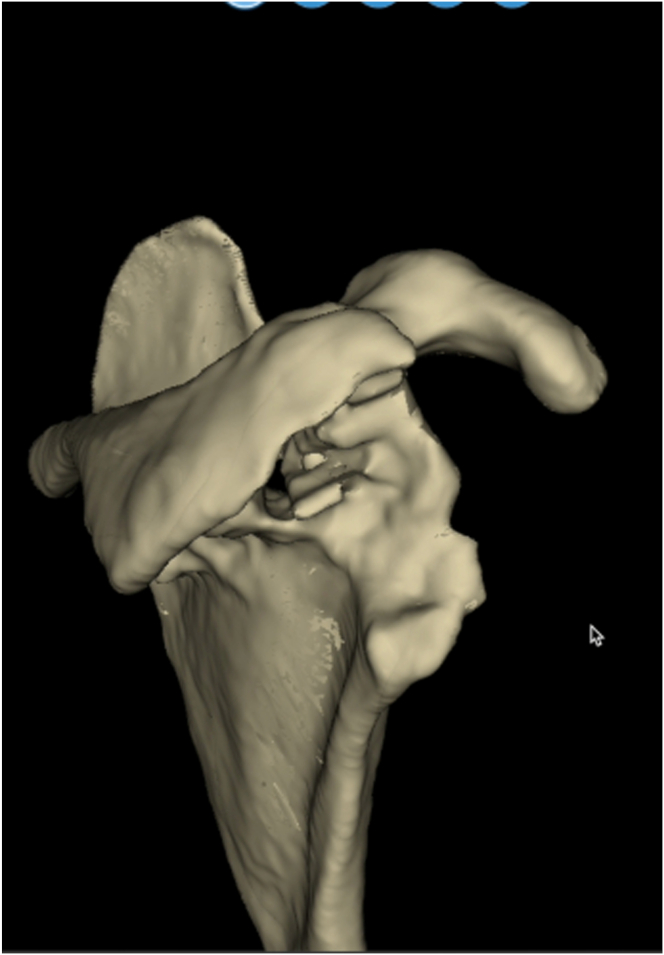
Three-dimensional model of the scapula illustrating the glenoid defect.

**Fig 3:**
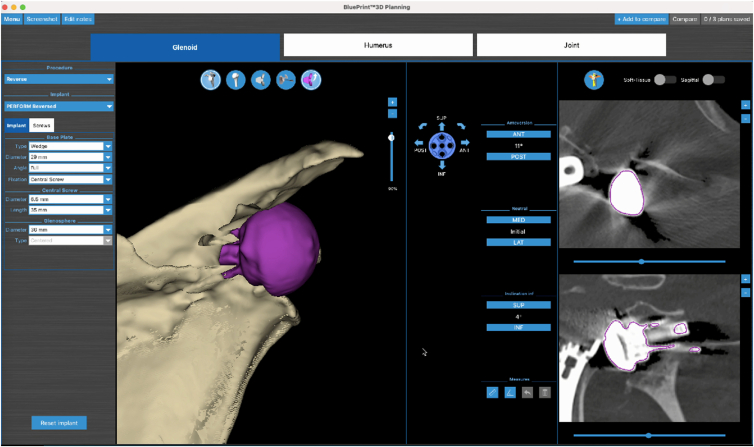
Essential preoperative 3-dimensional planning for management of severe glenoid bone loss.

Radiographic imaging (Fig 1) revealed loosening of the glenoid baseplate, glenoid dislocation, and possible glenoid fracture. Computed tomography revealed the presence of a severe posterior glenoid defect and a smaller one in the anterior wall (Fig 2). Comprehensive preoperative imaging was essential for accurate assessment and surgical planning. Three-dimensional computed tomography with virtual planning software aided in evaluating glenoid morphology and classifying bone defects (Fig 3). Table 1 describes the indications and contraindications for the sandwich technique in glenoid reconstruction.

**Table 1:** Indications and Contraindications for the Sandwich Technique in Glenoid Reconstruction

Indications	Contraindications
Severe anteroposterior glenoid bone loss	Previous Latarjet or Bristow procedure
Global bone loss requiring robust fixation	Insufficient iliac crest or coracoid size
Poor native glenoid bone quality	Advanced infection or osteomyelitis

### Surgical Approach and Glenoid Exposure

A deltopectoral approach is used upon reopening of the previous incision. The previous wound is removed with this standard incision beginning at the tip of the coracoid process extending distally. Aerobic and anaerobic samples are taken for microbiological analysis. The cephalic vein is preserved and retracted laterally. The subscapularis tendon, if still intact, is released via tenotomy to allow adequate glenoid exposure. A circumferential capsular release is carefully performed in order to protect the axillary nerve inferiorly. The anterior and posterior walls of the glenoid are meticulously cleaned to ensure complete visualization of the defects without soft tissue interference.

**Fig 4:**
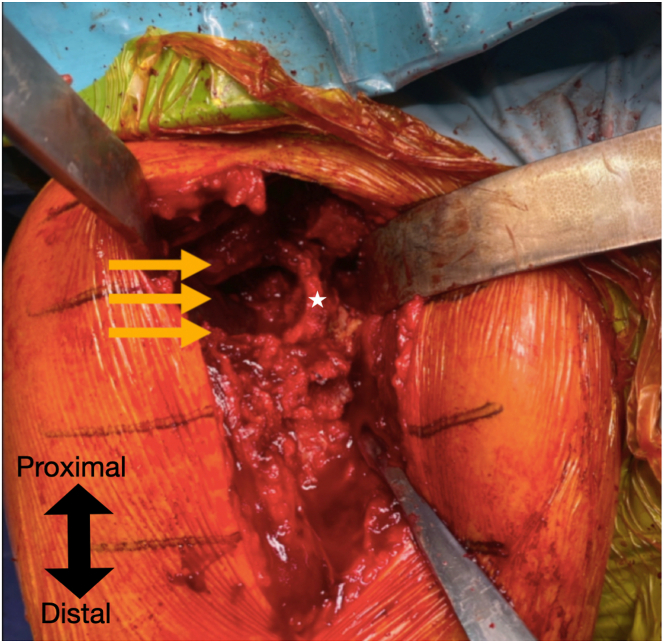
After removal of the previous implants, a severe posterior glenoid defect is observed (yellow arrows). The anterior glenoid wall remnant is indicated by the white star.

To optimize glenoid exposure, the humeral metaphysis and stem are removed, and the dislocated glenosphere and baseplate are extracted, revealing complete destruction of the glenoid (Fig 4). The primary finding in the glenoid is a posterior defect extending from the 12-o’clock to the 6-o’clock position, measuring approximately 25 mm in length and 7 mm in width (Video 1). The anterior defect is smaller, extending from the 3-o’clock to the 6-o’clock position, with dimensions of 9 mm in length and 4 mm in width. The proximal-to-distal length of the posterior defect is measured to determine the required dimensions of the iliac crest graft.

### Graft Harvesting

#### Iliac Crest Graft

A tricortical iliac crest graft of appropriate dimensions is harvested through a separate incision over the anterolateral iliac crest. The graft is meticulously shaped to fit the posterior glenoid defect and compensated with the glenoid anatomy, ensuring a flat surface for graft-to-bone contact. The cortical surface provides structural integrity, while the cancellous portion enhances integration.

#### Coracoid Graft

A coracoid osteotomy is performed at its base using a curved osteotome (Video 1). Soft-tissue attachments, including the conjoined tendon, are preserved to maintain vascularity. The graft is then rotated 90°, contoured to fit the anterior glenoid defect.

### Sandwich Grafting Technique

**Fig 5:**
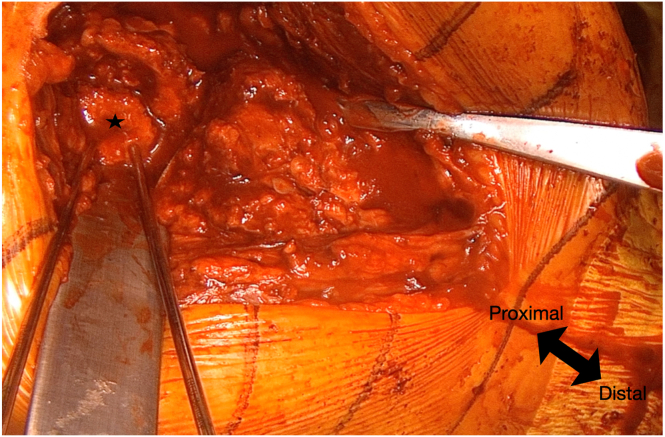
The graft is stabilized using 2 K-wires, one positioned superiorly and one inferiorly. The tricortical iliac crest autograft is indicated by the black star.

The sandwich grafting technique involves impacting the tricortical iliac crest graft with gentle pressure and stabilizing it in the posterior glenoid, initially securing it with 2 K-wires to create a flat, smooth surface (Video 1, Fig 5). The native glenoid remnant serves as the central layer of the construct. Anteriorly, the coracoid graft is fixed in the anterior defect below the glenoid equator with 2 K-wires, ensuring proper alignment with the native glenoid curvature.^13^ These 2 K-wires simultaneously stabilize the tricortical crest autograft as they are directed toward the tricortical iliac crest.

**Fig 6:**
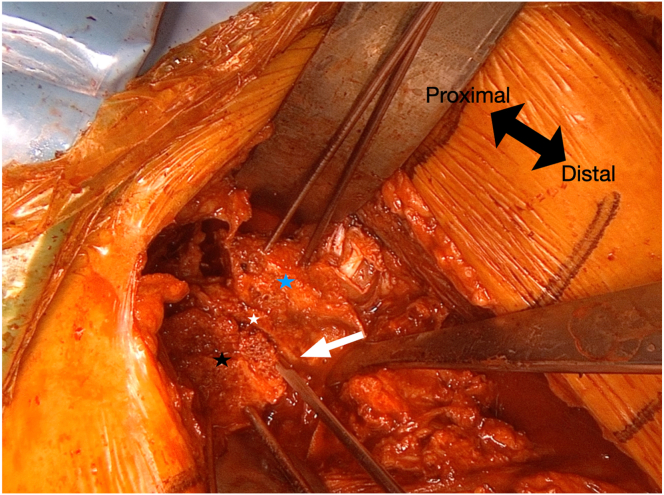
The glenoid baseplate guide (white arrow) is positioned at the central and inferior aspect of the glenoid. The coracoid process (blue star), anterior glenoid wall remnant (white star), and the tricortical iliac crest autograft (black star) are clearly visualized, comprising the key components of the "sandwich technique" construct.

**Fig 7:**
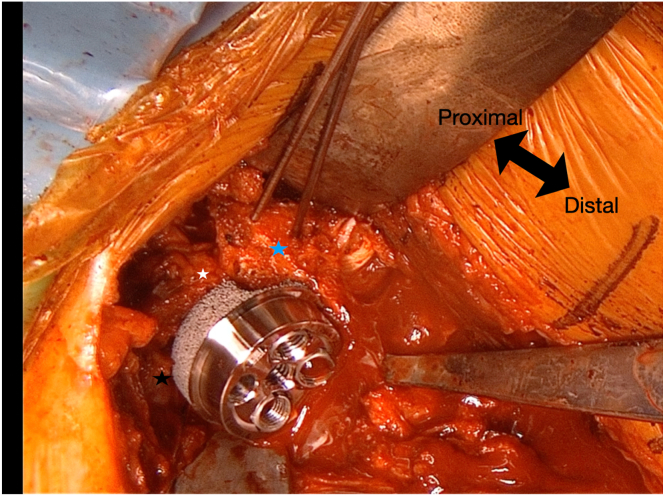
In the anteroposterior axis, half of the baseplate is positioned over the tricortical autograft, while the other half rests on the glenoid remnant and coracoid graft. This configuration enables secure fixation of the posterior graft with two screws. The central peg hole is drilled into the glenoid remnant, and a 15° full-wedge augmented baseplate with a central screw is positioned at the glenoid. The coracoid process (blue star), anterior glenoid wall remnant (white star), and tricortical iliac crest autograft (black star) are indicated.

Using the baseplate K-wire guide, a K-wire is placed inferiorly, flush with the inferior border of the glenoid (Fig 6). In the anteroposterior axis, half of the baseplate is positioned over the tricortical autograft, while the other half is placed over the glenoid remnant and coracoid graft (Fig 7). This technique allows for secure fixation of the posterior graft with 2 screws. The central peg hole is then drilled into the glenoid remnant, and a 15° full-wedge augmented baseplate (Perform Reverse; Stryker, Bloomington, MN) with a central screw is positioned at the glenoid.

**Fig 8:**
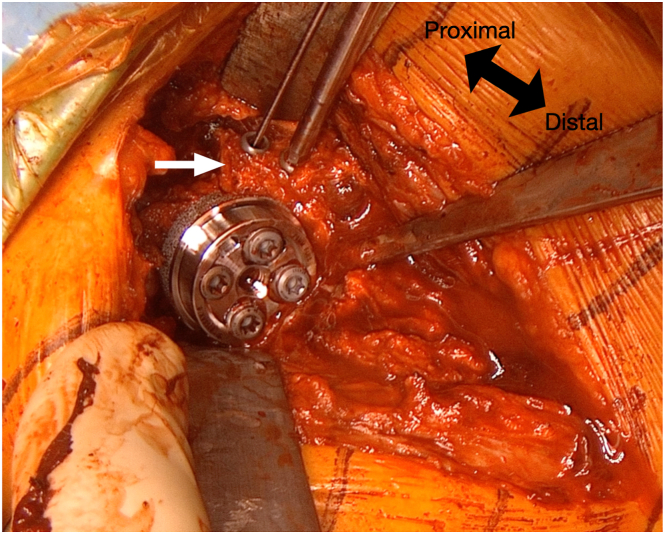
The coracoid process is further stabilized using 2 cannulated 4.5-mm partially threaded anterior screws (white arrow). Both screws are directed to engage the posterior tricortical iliac crest autograft, providing additional structural support to the construct.

**Fig 9:**
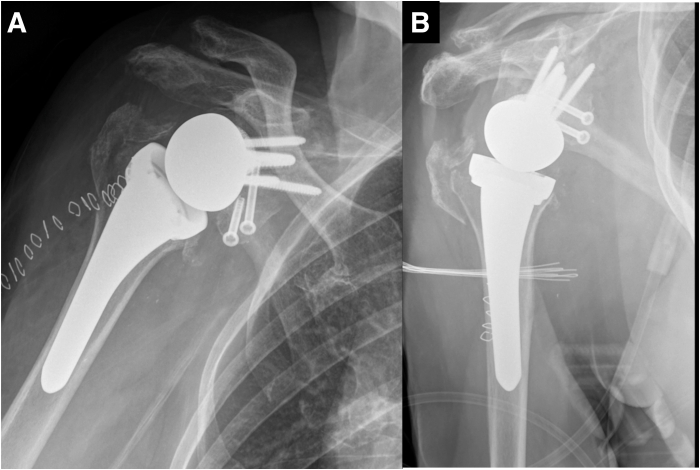
Postoperative anteroposterior (A) and lateral (B) radiographic views of the right shoulder, demonstrating the final position of the baseplate, screws, coracoid graft, and posterior tricortical iliac crest autograft.

**Fig 10:**
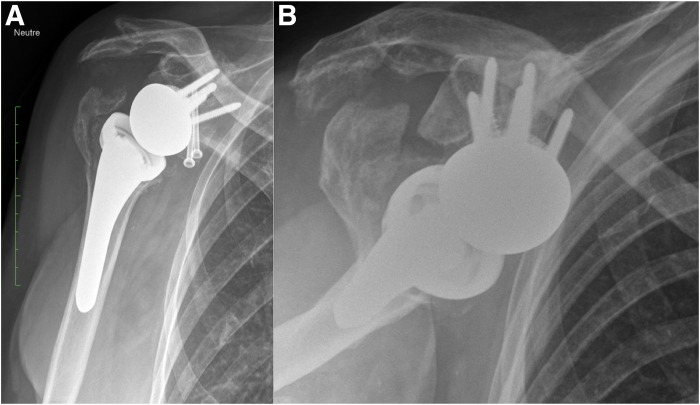
Anteroposterior (A) and lateral (B) radiographic views of the right shoulder at 9 months postoperatively, demonstrating maintained position and integration of the baseplate, coracoid graft, and posterior tricortical iliac crest autograft.

The first inserted screw is the superior cortical screw, which compresses the baseplate against the glenoid. The remaining 3 screws are locking screws, with the posterior and inferior screws further stabilizing the tricortical autograft. Next, two 4.5-mm partial-threaded anterior screws are inserted to stabilize the coracoid (Fig 8). Both screws are also directed to engage the posterior tricortical iliac crest autograft for additional support. Afterward, the 2 K-wires stabilizing the iliac crest graft are removed. The wedge design provides inferior tilt, converting shear forces into compressive forces to enhance fixation and graft integration. Screws positioned in 2 axes ensure stability of the baseplate in both the anteroposterior and mediolateral directions. Finally, a 36-mm glenosphere is implanted (Perform Reverse; Stryker) and a short stem (Medacta Shoulder System; Medacta, Castel San Pietro, Switzerland) is implanted (implant mismatch).^14^ Surgical pearls and pitfalls and advantages and disadvantages of this technique are summarized in Tables 2 and 3, respectively. Figures 9 and 10 show radiographs immediately postoperative and at 9 months, respectively.

**Table 2:** Surgical Pearls and Pitfalls for the Sandwich Grafting Technique

Pearls	Pitfalls
Use a full wedge baseplate for enhanced stability	Risk of improper wedge positioning
Harvest iliac graft with sufficient cortical support	Risk of graft fracture during screw placement
Ensure proper orientation of coracoid graft	Incorrect alignment leading to instability
Drill multiple holes in glenoid to enhance integration

**Table 3:** Advantages and Disadvantages of the Sandwich Technique

Advantages	Disadvantages
Combines vascularized and structural grafts	Technically demanding
Improves graft integration rates	Increased operative time
Enhances fixation and stability	Potential morbidity from iliac crest harvesting
Reduces risk of graft lysis	Size of coracoid graft may limit applicability
Benefits from full-wedge baseplate design	Requires specialized instrumentation

### Closure and Postoperative Protocol

If possible, the subscapularis is reattached with nonabsorbable sutures^15^ and eventually reinforced^16^ to restore anterior stability and functional internal rotation.^17^ The presence of a graft behind the subscapularis, even without subscapularis split, is not known to induce symptomatic impingement.^13^^,^^18^ A layered closure is performed, ensuring adequate soft-tissue coverage. Postoperatively, the arm is immobilized in an abduction pillow for 6 weeks. Passive range-of-motion exercises begin afterward, with gradual progression toward active movements to restore function and optimize outcomes.

## Discussion

The sandwich technique is indicated for patients with severe anteroposterior or global bone loss based on Walch classification types B3, C, and D, when standard reconstruction methods are inadequate. This technique integrates biological and mechanical fixation by using a structural autograft, a vascularized autograft, and a metallic baseplate. The vascularized coracoid graft promotes healing and integration, whereas the iliac crest graft provides a stable, rigid foundation for the glenoid construct. The full-wedge baseplate enhances stability by converting shear forces into compressive forces. This dual-graft approach effectively addresses the challenge of achieving stable fixation in cases in which single-graft techniques or augmented baseplates are inadequate.

Glenoid bone grafting in the setting of conversion to RSA has an overall failure rate of 18% regardless of graft type, whether autograft or allograft. Fliegel et al.^19^ underscored this limitation in their review on biologic graft augmentation for glenoid bone loss during the conversion of failed anatomic shoulder arthroplasty to RSA. Similar findings were reported by Malahias et al.^20^ in their review, indicating that the all-cause implant re-revision rate for revision RSA with glenoid bone grafting was 21.1%.

Stability is the key advantage of this technique. The anteroposterior orientation of the screws securing the iliac crest and coracoid grafts creates a compressive force across the sandwich construct, enhancing mechanical interlock. In addition, the screws of the baseplate are strategically angled to cross from lateral to medial, providing superior stabilization by integrating the grafts and the native glenoid. The full wedge augmented baseplate further contributes to this stability by transforming shear forces into compressive ones.^21^ The fixation screws positioned in both the anteroposterior and mediolateral axes further reinforce construct stability which is critical for promoting graft healing and integration.

Another major advantage of the sandwich technique is the reduced risk of graft lysis. In contrast to allografts, which have a greater incidence of resorption and lysis because of their lack of vascularity, this method employs autografts that integrate more effectively with the host bone.^11^ The vascularized coracoid graft further enhances this integration, reducing the risk of resorption and ensuring a more durable reconstruction.

The incorporation of the full wedge baseplate offers additional advantages over traditional baseplate designs. By providing a large contact area and inferior tilt, the wedge design ensures greater stability and minimizes micromotion at the graft-baseplate interface. These features, as highlighted in recent literature, make it particularly effective for cases of severe glenoid bone loss.^22^, ^23^, ^24^ Limitations are discussed in the Tables 2 and 3.

The sandwich grafting technique provides a biomechanically stable and biologically integrative solution for severe glenoid bone loss. By combining structural and vascularized autografts with a wedge-augmented baseplate, this method enhances fixation, promotes graft incorporation, and restores native glenoid anatomy.

## Declaration of generative AI and AI-assisted technologies in the writing process

During the preparation of this work the author(s) used ChatGPT in order to improve grammar, syntax, and clarity of the relevant section. After using this tool/service, the author(s) reviewed and edited the content as needed and take(s) full responsibility for the content of the publication.

## Disclosures

The authors declare the following financial interests/personal relationships which may be considered as potential competing interests: A.L. is a paid consultant for Arthrex, Stryker, Medacta, and Enovis; received royalties from Stryker and Medacta; is the (co-)founder of FORE, Med4Cast, and BeeMed; owns stock options in Follow Health; is on the board of the French Arthroscopic Society. P.C. reports that he receives royalties from and is a consultant and paid speaker for Stryker and Enovis; is the co-founder of Med4Cast and Follow and is on the board of SECEC and IBSES. All other authors (V.A., G.L., J.Z.) declare that they have no known competing financial interests or personal relationships that could have appeared to influence the work reported in this paper.

## Supplementary Data

Video 1 The sandwich technique for reconstruction of severe glenoid defects. This video demonstrates the sandwich technique for managing severe glenoid bone loss. The procedure begins with harvesting a tricortical iliac crest autograft and the coracoid process through a deltopectoral approach. After implant removal, a significant posterior glenoid defect is visualized. The posterior defect is reconstructed with the tricortical autograft, impacted and stabilized with 2 K-wires. The coracoid is transferred anteriorly and fixed with 2 additional K-wires. A baseplate guide is positioned centrally and inferiorly on the glenoid, the peg hole is drilled, and the baseplate is placed. Screws are inserted for fixation, and the coracoid is further secured with two 4.5-mm cannulated screws engaging the posterior graft. Final steps include glenosphere placement, stem insertion, liner impaction, and joint reduction. The video concludes with postoperative radiographs and 9-month follow-up, confirming stable integration of all grafts and components.
